# Local tips, global impact: community-driven measures as avenues of promoting inclusion in the control of neglected tropical diseases: a case study in Kenya

**DOI:** 10.1186/s40249-022-01011-w

**Published:** 2022-08-05

**Authors:** Elizabeth A. Ochola, Diana M. S. Karanja, Susan J. Elliott

**Affiliations:** 1 Department of Geography and Environmental Management, University of Waterloo, Waterloo, ON Canada; 2 COHESU, West Kajulu, P.O. Box 2956-40100, Kisumu, Kenya

**Keywords:** Neglected tropical diseases, Control, Community-driven measure, Local solution, Global impact, Inclusion, Kenya

## Abstract

**Background:**

Neglected tropical diseases (NTDs) affect poor populations with little or no ‘political voice’ to influence control activities. While most NTDs have interventions that work, the biggest challenge remains in delivering targeted interventions to affected populations residing in areas experiencing weak health systems. Despite the upward development trends in most countries of sub-Saharan Africa (SSA), the healthcare worker to population ratio remains exceptionally low, with some areas not served at all; thus, there is a need to involve other personnel for school and community-based healthcare approaches. Nonetheless, the current community-based programs suffer from inconsistent community participation due to a lack of coordinated response, and an expanded intervention agenda that lacks context-specific solutions applicable to rural, urban, and marginalized areas.

**Methods:**

This research investigated the capacity of local communities to address the burden of NTDs. Informed by the social theory of human capability, the research collected primary qualitative data by conducting key informant interviews and focus group discussions of people infected or affected by NTDs. The interview data were collected and transcribed verbatim for thematic analysis using Nvivo version 12.

**Results:**

Our findings reveal, first, a need for intersectoral collaboration between governments and affected populations for inclusive and sustainable NTD solutions. Second, a ‘bottom-up’ approach that enhances capacity building, sensitization, and behaviour change for improved uptake of NTD interventions. Third, the enforcement of Public Health Legislative Acts that mandates the reporting and treatment of NTDs such as leprosy. Fourth, the establishment of support groups and counseling services to assist persons suffering from debilitating and permanent effects of NTDs.

**Conclusions:**

Our research demonstrates the importance of human agency in encouraging new forms of participation leading to the co-production of inclusive and sustainable solutions against NTDs.

**Supplementary Information:**

The online version contains supplementary material available at 10.1186/s40249-022-01011-w.

## Background

Neglected tropical diseases (NTDs) are a cumulative group of 20 diseases and conditions that are etiologically, epidemiologically, and clinically linked to poverty [1, 2]. They collectively affect more than one billion people causing devastating health, social and economic consequences on individuals, families, and communities.

The Berlin meetings of 2003 and 2005 set the pace for global NTD control initiatives [2] by influencing the World Health Assembly (WHA) to come up with speedy resolutions for resource mobilization, drug donations, and the improvement of delivery mechanisms for integrated control programs [3]. Through this recommendation, pharmaceutical companies, governments, non-governmental organizations (NGOs), and public health professionals saw the need to have a ‘moral investment’ towards NTDs by establishing measures that positively impact the lives of people and reflect on their choices in the kind of life they would like to live [4, 5].

Four decades ago, smallpox was eradicated in the public health arena and with it came long-lasting health benefits to society. For example, it was realized that significant improvements in people’s health status could be improved using small budgets and well-trained health staff when clear steps were taken to involve residents [6]. Similarly, the dracunculiasis eradication program has facilitated a decline in global cases from approximately 3.5 million cases in the mid-1980s to about 54 human cases in 2019 [7]. Most countries previously endemic to dracunculiasis are in the certification process, with countries like Kenya having eradicated the disease in 2017 [8].

The current knowledge generated in years of research demonstrates that minimum diagnostic tools and strategies are available to assess the distribution of NTDs and inform control, elimination, and eradication programs on a global, national, regional, and local scale [4, 9, 10]. As such, the World Health Organization (WHO) recommends that intervention activities be applied along NTD ‘hotspots’ [11, 12] with the necessary political, economic, and social support [4, 13, 14].

Since 2010, significant improvement has been made in combating NTDs in sub-Saharan Africa (SSA) as a result of the 2008–2015 roadmap [15, 16]. There have been numerous community partnerships that aim to promote equitable and effective NTD control strategies [17]. However, context-specific solutions remain crucial in designing and implementing community directed intervention (CDI) strategies in rural, urban, and marginalized areas. For example, the African Programme for Onchocerciasis Control (APOC) was among the first successful strategies that utilized CDI to distribute ivermectin [18, 19], demonstrating the need for community participation and ownership. Similarly, Wanji et al. [20] and the World Bank [21] report that community members involved at the onset of onchocerciasis interventions appreciated their engagement as they were able to take part in the selection of community health implementers, which built trust and was a motivator for increased uptake of NTD activities in the community. For this reason, successful primary health care (PHC) strategies are rooted in communities that allow community members to make or influence decisions that affect their health [22].

Most NTDs have available interventions that work; the most significant challenge remains how to deliver interventions to affected populations in areas experiencing weak health systems [23, 24]. Community directed treatment (CDT) approaches provide an avenue for health providers to work closely with community members for effective healthcare delivery in specific settings [22, 25, 26]. The CDT approach involves selecting and training community health volunteers (CHVs), enabling community members to decide on the intervention locality and how monitoring and supervision will be done [4]. In as much as CHVs are shown to have a wealth of knowledge, are capable and reliable, they require incentives to remain motivated and support intervention strategies; hence, the focus of action needs to go beyond health worker engagement towards collective action and responsiveness of the health systems to the needs and realities of individuals [4, 27]. Health equity is a fundamental human right at the centre of universal health care (UHC) which aims to ensure that all persons have equitable and barrier-free access to healthcare regardless of their social and economic status [28]. The integrated people-centred health services (IPCHS) propose catering to the holistic needs of individuals and communities by empowering them to be active in determining and satisfying their health needs [22, 28].

The persistence of NTD infection is attributed to poverty and structural inequity [29], which affects the distribution of healthcare resources since social policies and resources are connected to wealth, power, and prestige, which create barriers for marginalized populations. Whereas public health interventions require political action to have sufficient health policies in place, the role of collective action remains equally important. For example, Shiffman and Smith [30] explore the role of collective action in harnessing the power to encourage action for global health initiatives. Comparably, Hussaarts et al. [31] find that Research and Development (R&D) programs for NTDs integrate resources, technical facilities, and public–private partnerships to collectively develop and deliver safe and affordable treatments to affected populations.

Bisung et al. [32] emphasize the importance of social capital to spur collective action since it predisposes people towards cooperative behaviour necessary to facilitate shared goals. The mobilization of activities at the local level provides an opportunity for the use of collective action [9, 33] since intersocial networks increase information uptake through solidarity, cultural change, and disease prevention [34, 35]. However, it remains challenging to sustain NTD programs given that issues such as social perception, supervision, and surveillance persist on the ground. Hence the need for consistent advocacy at the local level to maintain momentum [5, 36, 37]. For this reason, our research explored the capacity of Kenyan communities to address the burden of NTDs. It specifically examined the role of collective action as a means by which community members can identify the kind of sustainable contributions they can make toward NTD control.

### Theoretical framework

Theory provides an opportunity to explain processes in the environment, socio-economic systems and understand the importance of agency [38–41]. The capability theory is a broad and normative social framework that assesses individual wellbeing, policy design, and societal changes [42, 43]. Developed by Amartya Sen, the approach consists of the key concepts of capability, functioning, and agency [44, 45]. There is no definitive list of functioning because different things are relevant to different people in different settings [46]. A person’s capability entails a combination of functionings [45], depending on opportunities. Thus, capability reflects freedom, opportunities, human rights and is a powerful concept to use when addressing poverty, injustice, and wellbeing [46, 47]. In addition to capability and functioning, there is agency which evaluates “what a person can do and achieve in pursuit of whatever goals or values he or she regards as necessary” [48].

The capability approach acknowledges that poverty and inequity can constrain freedom, choices, and agency [49]. Moreover, a deprivation of capabilities affects access to health care, education, participation in the economic system, and a lack of autonomy among women [50, 51]. Hence the capability theory provides a unique lens to address the complexity of infectious diseases such as NTDs since poverty causes a deprivation of freedom, capabilities and further impairs opportunities and choices in life [52]. As such, there is a need to move away from the established paradigm of vertical NTD programmes and shift the power structures and decision-making from governments to communities to provide an avenue for individuals to frame their problems and generate knowledge and solutions that help them advocate for themselves [53] in an empowered and sustainable manner.

## Methods

### Study context

The study was conducted in Kenya, which lies on the east coast of Africa, an area endemic for lymphatic filariasis (LF), schistosomiasis, soil-transmitted helminths (STHs), trachoma, among other NTDs [54]. The Ministry of Health guides the NTD activities in the country through the National Strategic Plan for Control of Neglected Tropical Diseases (2016–2020) [55, 56]. One of the plan’s guiding principles is to scale up access to interventions, and treatments and build capacity for a productive nation free from NTDs. As such the study was conducted in five NTD endemic counties: Busia, Kilifi, Kisumu, Nairobi, and Turkana. All the areas selected were endemic for more than one NTD.

### Ethical consideration

The research protocol was approved by the University of Waterloo Research Ethics Committee (ORE#22493) and the Maseno University Ethical Review Board (MSU/DRP/MUERC/00496/17) to satisfy the requirements of conducting research studies in Kenya. Written consent was obtained before the interviews or focus group discussions were initiated, and the participants were informed of their right to decline participation or answer questions they deemed uncomfortable. The participants were also informed that they could stop their interaction with the research team at any point.

### Research design

The study was qualitative, and it used purposive sampling to conduct key informants (KIs) interviews and focus group (FG) discussions to understand the capacity of Kenyan communities to address the burden of NTDs. Purposive sampling ensured a maximum variation and validation across the different characteristics by age, geographical composition, and socio-economic status. In our research context, key informants were regarded as directly engaged with the Kenyan health sector, considered knowledgeable on NTD issues, and understood the barriers to NTD interventions. Furthermore, the use of KIs provided an insight into the current policy aspects of NTD control that were not easily obtained by other forms of data collection.

Focus group discussions involved interviewing the participants in a group setting to facilitate collective dialogue. The use of FGs enabled the participants to freely discuss their experiences in a supportive environment [57]. The group dynamics allowed participants to provide their perspectives as well as listen to the thoughts of others and develop potential solutions.

### Data collection

Before fieldwork commenced, there was a meeting with the Ministry of Health officials, health workers, local NTD partners, County officials, village elders, and community members to discuss the general purpose and objective of the research. During the meeting, individuals were asked to share their thoughts and ask questions. Later on, the consent process, potential risks, benefits, privacy, and confidentiality concerns were addressed.

The KIs and FGs discussants were contacted either in person, by telephone, or by email and provided with information letters that outlined the research objectives. Data collection took three months, from November 2017 to February 2018. The interviews were conducted using an interview guide (Appendix 1) informed by the constructs of the capability approach to understand the current intervention strategies and explore how the community can address the burden of NTDs. The use of interview guides and probes ensured the discussions were flexible, and the use of pseudonyms ensured confidentiality.

A total of 21 KIs and 46 individuals participated in the FGs, respectively; the participants were aged between 18 and 61 years. The KI interviews lasted between 30 and 90 min while the FGs lasted between 45 and 90 min. Both interviews were conducted either indoors at an office or outdoors in quiet locations. All the participants’ responses were effectively captured, and probes were used to seek clarification and enhance accuracy and validity [58].

The interview data was collected until a point where no new data emerged (saturation). The use of theoretical saturation in the research allowed for the examination of the phenomenon predicted to fit within or outside the original theoretical constructs. The use of theoretical saturation also allowed an openness for data that is external to the theory to emerge, thus identifying non-conforming cases and exploring the extent of the differences [59].

### Data analysis

The audio recording from the interviews was transcribed verbatim for thematic analysis using Nvivo version 12 (QSR International Ltd, Burlington, Massachusetts, USA). The transcripts were first scanned to determine codes and form a coding manual. The codes were created using a deductive approach that explored data related to the theoretical construct of the capability approach and the research objective (to investigate the capacity of local communities to address the burden of NTDs). An inductive approach determined emerging themes from the data. Next, the data was coded line-by-line to produce textual elements later organized into themes and sub-themes [60].

Once the coding manual for each participant group was completed, inter-rater reliability [61, 62] was used to enhance scientific rigour. For each interview, two transcripts were coded by the first author and a second researcher. The coding agreement was pegged at greater than 75%. The emerging coding differences were discussed, reconciled, and used to revise the coding manual for the remaining transcripts. The use of this process facilitated intercoder reliability through comparison and further strengthened the credibility of the results [61].

The results were organized around the research objective, which was to investigate the capacity of local communities to address the burden of NTDs. The findings were summarized in Tables. The factors that mattered most to the participants were based on the frequency of mentions (the number of times an indicator was mentioned within the categories) and the number of participants who mentioned the category in both groups (KIs and FGs), direct quotations were also used to punctuate the themes (Additional file 1). Each quote was identified by a pseudonym, gender, age, and the data collection technique (Additional file 1). The results section highlights the current NTD programs in Kenya, followed by the proposed interventions from the communities (FGs) and the proposed NTD interventions or policies from the KIs.

## Results

### The current NTD programs in Kenya

The most frequently mentioned and discussed factor by KIs and FGs was the school-based deworming program carried out by the Government of Kenya and its partners (Table 1). For example, the participants affirmed that the deworming program had decreased worm infestations and increased school participation.

**Table 1:** The current NTD programs in Kenya

	Key informants (*n* = 21)		Focus groups (*n* = 5) Total participants (46)	
	No. of KIs	No. of mentions	No. of FGs	No. of mentions
Current NTD programs				
School-based deworming program	11	28	5	25
Community-Led Total Sanitation (CLTS)	10	19	5	34
WASH interventions • Chlorine filters • ‘Unilever School of 5’	10	16	4	25
National school health policy	4	3	NM	0
Program assessment for onchocerciasis and Human African Trypanosomiasis	1	3	NM	0
Active case findings	1	1	NM	0

*NTD* neglected tropical diseases, *FGs* focus groups, *NM* not mentioned, *KIs* key informants

Our data revealed that most counties adopted the community-led total sanitation (CLTS) program to prevent open defecation and reduce the transmission of worms. A county being CLTS compliant meant that everybody had access to a toilet or latrine. At the community level, the FG participants revealed that WASH interventions (Table 1), which included providing safe drinking water through chlorine filters installed in water collection kiosks, were commonly available at the water source. While at the school level, WASH interventions included personal hygiene, face washing, and the ‘Unilever School of 5’ which focused on handwashing at five critical times: when somebody woke up, before eating, after eating, after using the toilet, after blowing the nose or coughing and programs like trachoma control were using this approach to promote eye health. Other ongoing programs (Table 1) included establishing and reviewing the National school health policy, which was solely discussed by the KIs. The National school health policy has eight thematic areas that guide WASH and deworming activities.

Our analysis indicated that in 2018, Kenya embarked on a program assessment for onchocerciasis & human African trypanosomiasis (HAT) to begin the certification process for the two NTDs. It was also revealed that there were ongoing case-finding activities for leprosy and hydatid disease, as well as future plans to operationalize the NTD database to capture real-time disease prevalence.

### The proposed interventions from the communities

The community members discussed that counseling services and support groups for persons infected with long-term disabling NTDs such as leprosy, elephantiasis, trachoma, and snake bites were lacking (Table 2). Our respondents noted that such support groups could be avenues for information sharing similar to HIV/AIDs support groups*.*

**Table 2:** The proposed NTD interventions from the communities

Proposed interventions from communities		
	Focus groups (*n* = 5) Total participants (46)	No. of mentions
Counselling services and support groups	5	15
Early physical examination	5	7
Proper diagnosis	4	13
Adequate health worker to patient numbers	4	9
Community sensitization	4	9
Political goodwill	4	7
Improvement in WASH facilities	4	9
Access to care, treatment, and UHC	4	7

*NTD* neglected tropical diseases, *UHC* universal health coverage, *WASH* water, sanitation, and hygiene

The importance of early physical examination was discussed specifically for conditions such as hydrocele to capture the swelling as a result of LF infection at an early stage. The community members also discussed the importance of proper diagnosis of NTDs at the health facilities (Table 2), and the availability of an adequate number of health care workers to enable prompt treatment within their locality. Our data also revealed a need for continuous sensitization to minimize ignorance as well as continuous political goodwill from the county governments as an avenue for community empowerment.

Other discussions were on the improvement of WASH facilities such as the provision of piped water, water treatment facilities, and the construction of latrines at the household level. At the health facility level, the community members proposed that access to care and treatment (Table 2) be provided free of charge or at an affordable rate as Kenya works toward providing UHC to its citizens since the current National health insurance fund (NHIF) remains unaffordable to many.

### The proposed NTD interventions/policies from key informants

Our findings revealed that NTD interventions coordinated by the Ministry of Health, the Ministry of Education, County governments, and other stakeholders need to be integrated (Table 3).

**Table 3:** Proposed interventions from key informants

Proposed Interventions from Key informants	Key informants (*n* = 21)	
	No. of Key informants	No. of mentions
Intersectoral collaboration	7	25
Bottom-up’ capacity building	6	22
Enforcement of NTD legislative acts	5	12
Sensitization and behaviour change	4	17
Counselling and support services	4	6
Improved vaccination services	4	5

*NTD* neglected tropical diseases

For example, KIs proposed collaboration within the ministries and County governments and also between the ministries and the affected populations. For example, they discussed NTD integration with other sectors such as housing, agriculture, and veterinary.

There was a need to engage communities through capacity building around NTD control. This could involve forming active community committees comprising health workers, CHVs, and individuals in the community for health education and NTD surveillance. Capacity building through a bottom-up approach that involves not thinking for the community but getting the community to give ideas on policy formulation and what they want these policies to address.

The KIs suggested that legislative acts (Table 3) around the treatment of NTDs need to be enforced for highly infectious NTDs. For example, section 84 of the Public Health Act CAP 242 states that persons receiving treatment for leprosy need to be followed up to ensure that they adhere to the treatment regimen. The Act further states that when a person does not present at the health facility to take medication, then the law mandates the public health officer to follow up with the person and ensure they adhere to the treatment. Furthermore, NTDs need to be included in the school and public health curriculum for sensitization and behavioral change among young adults.

The KIs proposed that individuals suffering from long-term debilitating and disabling NTDs such as leprosy, elephantiasis, trachoma, and snake bites required counseling services and support groups similar to the HIV/AIDS and diabetes support groups (Table 3) to help them deal with the psychosocial effects of NTDs. The KIs further discussed an improvement in vaccination services, with NTD vaccines such as rabies being part of the Kenya expanded programme on immunization (KEPI) rather than being on-demand vaccines when the need arises.

## Discussion

The research used the social theory of capability to investigate the capacity of local communities to address the burden of NTDs. For a long time, NTD programmes were driven by external actors and institutions that reinforced a top-down paradigm for infection control at the expense of socio-ecological factors that underlie transmission cycles [53]. The use of such vertical programs often lacked community ownership and trust, which are essential elements of NTD control in the face of dynamic power structures [53, 63, 64]. Despite such barriers, it remains equitable, progressive, and successful to have interventions that are designed and implemented within the community by active rather than passive members [17, 18].

In 2013, WHA adopted a resolution to intensify efforts toward eliminating and eradicating NTDs by integrating them within primary health services and the SDGs for a strong call to action [65]. In Kenya, the current NTD control initiatives include community-run programs such as CLTS and WASH, while at the national level, there are interventions such as school-based deworming programs and program assessments. The WHO 2030 strategy on ending the neglect caused by NTDs to attain the SDGs is a call to action with crucial shifts: strengthening cross-cutting programs to include all persons and supporting patient-centred holistic approaches. Moreover, the current roadmap aims to change operating models away from partner-led initiatives toward country-driven and country-owned programming that reflects the realities of the local people [14, 36]. As such, this study applied human agency to explore community-driven measures against NTDs.

First, our results indicated a need for intersectoral collaboration at the national level, meaning integration within and between ministries and the affected populations. A case example is the Ministry of Water providing WASH facilities, the Ministry of Agriculture empowering communities on alternative economic activities that promote food security and livelihoods, the Ministry of Veterinary Services providing sustainable vector control practices, the Ministry of Public Works exploring ways in which infrastructure can be improved by building better roads, bridges and proper drainage systems and in all this, engaging the political class at the County governments for implementation and supervision. Similarly, the WHO 2030 roadmap advocates for cross-sectoral strategies such as the One Health approach towards zoonotic NTDs, vector management, and environmental health to sustain the gains that have been made [14]. The use of such integrative approaches encourages local partnerships that advocate for holistic benefits beyond health towards wellbeing for individuals living in NTD endemic areas [4]. Furthermore, our results revealed the need for policy action that complements community messaging to ensure that health systems cater to the needs and realities of citizens [4, 27]. Community-based approaches have been proven to have the greatest impact on health outcomes in areas that have weak health systems and higher incidences of mortality and morbidity [27].

Second, our results confirmed that the ‘bottom-up’ approach enhances capacity building, sensitization, and behaviour change as catalysts for improved uptake of NTD interventions since they involve forming active community committees comprising of health workers, CHVs, and individuals for health purposes, education and NTD surveillance. Comparatively, Onasanya et al. [66] report that an active approach to NTD control in SSA requires dynamic and diversified bottom-up processes that involve multiple stakeholders and paves the way for the achievement of the NTD 2021–2030 goals. An increased social capital within the community and between the community and the national health system may lead to better health outcomes [27].

Third, was the enforcement of legislative acts, for example, CAP 242 of the Public Health Act (section 78) mandates the reporting of suspected leprosy cases in an area. The legislation ensures that a person suffering from leprosy voluntarily receives treatment, and adheres to treatment and follow-up procedures (section 84) [67]. At the local level, there is a need to have early case finding and proper diagnosis to capture NTDs such as leprosy in their early phase and avoid disability associated with the disease in the chronic stage. The importance of early physical examinations, proper diagnosis, and treatment cannot be overemphasized because most of the NTDs present as febrile illnesses (showing signs of fever) at their onset, for example, dengue, chikungunya, lymphatic filariasis, schistosomiasis, and they may progress to cause disability and even death. As a result, communities living in NTD endemic areas need access to proper diagnosis and treatment from their nearest health facilities as stipulated in the WHO policy document on primary health care [68].

Fourth, our results revealed the need to have support groups and counseling services to assist persons suffering from debilitating and permanent effects of NTDs such as leprosy, lymphatic filariasis, trachoma, and snake bites. The establishment of support groups will allow the individuals to share their experiences and develop coping skills, which will reduce stigma and discrimination against them. Furthermore, support groups improve self-esteem, and provide an avenue for adherence to care and medication. Support groups have successfully been used by persons living with HIV/AIDs [69, 70].

Currently, the cost of accessing health care is out of reach for many people, especially those living in marginalized areas; our findings suggest that the Government of Kenya should strive to provide UHC to all its citizens regardless of their socio-economic status [71, 72]. Furthermore, UHC should be extended to include rapid diagnosis and treatment of NTDs at affordable or no costs to the citizens of Kenya.

In regard to policy implications, we found that NTD infection in Kenya is a major problem that needs to be addressed as the country works towards achieving the WHO 2030 goals. Hence policymakers must work with communities to establish context-specific solutions that are sensitive to culture and behaviours. Our findings suggest that working with communities residing in endemic areas of Kenya is a sustainable avenue for promoting collaborative information exchange and increasing the uptake of NTD activities between affected communities and the different actors in the health system. By establishing complementary context-specific solutions, the affected populations are encouraged or better placed to advocate for NTD policy changes that are tailor-made to benefit them.

In our context, community involvement in NTD interventions inspired vulnerable communities to find their voice, define their participation, and ensured that they engaged with other actors at an equitable level. Building on existing health system structures, the communities that we interviewed were willing to be flexible in the uptake of NTD activities if it led to collective change that would end the neglect. We acknowledge that the previous extensive use of vertical programmes in NTD initiatives advanced power inequities in the delivery of health care, but we are encouraged that policy changes towards community-driven programs may motivate the co-production of NTD solutions leading to new forms of participation and exploration in health delivery. Thus, by engaging communities, we anticipate that NTD stakeholders will develop structures for ongoing health education across multiple scales leading to the co-creation of inclusive and sustainable solutions for NTD programs.

Despite the substantive contributions to policy, our research had limitations. First, the research was carried out when Kenya was experiencing political turmoil after the 2017 general elections. During this time, it was difficult to reach certain areas of the country due to political tension, but the sampled study areas provided a comprehensive outlook of the NTD situation in the country. Second, our study used purposive sampling to ensure a maximum variation and validation across characteristics i.e., age and gender among FG participants and engage KIs who were considered knowledgeable on current NTD policies. However, the use of these sampling techniques may have introduced some bias. To minimize this potential bias, we, captured elements of similarity between themes, depth of understanding, and theoretical saturation in interviews for both FGs and KIs. Third, our study was qualitative and thus based on self-reported data, which is susceptible to social desirability. In this case, the participants could overstate or understate their experiences depending on expectations. As such, we asked follow-up questions to the participants to minimize bias and used subtle probes to enhance the research discussions. Fourth, our study was crossectional, meaning the data was collected at a single time point which did not examine potential changes over time regarding the ongoing country-wide intervention strategies. However, we used a triangulation of methods (KIs and FGs) to ensure similarity in the emerging themes and minimize bias.

## Conclusions

Our research used the capability approach to investigate the capacity of local communities to address the burden of NTDs. Our results indicate a need for intersectoral collaboration, ‘bottom-up’ capacity building, enforcement of Public Health Acts, and the establishment of support groups and counseling services. Our use of social theory provided an explanatory power in the predominantly biomedical field of NTDs to encourage human agency and collective action for inclusive and sustainable solutions to NTDs in Kenya. Furthermore, by using the capability approach, we found that NTD infection deprives affected persons of freedom and opportunities; thus, we recommend community-driven measures that foster collaboration to ensure that suggestions from the affected community members are aligned with the broader structural goals.

### Supplementary Information

**Additional file 1.** Supplementary Tables and Appendix.

## Data Availability

All data generated or analyzed during this study are included in this published article [and its additional information files].

## Appendix 1

**Table:**

Interview guide for collecting data on the capacity of local communities to address the burden of NTDs	
Construct	Question
Neglected Tropical Diseases (NTDs)	Tell me about life in your community What about the health in your community? What are some of the Neglected Tropical Diseases (NTDs) that are prevalent in your community?
NTDs and health service provision	What makes for a healthy community? What are some of the important issues in health care provision? Would you consider the health care system effective? If yes/no, why? Can we talk about accessibility? Drug availability, health insurance, specialized care and waiting time? How have you adapted? Are there alternatives?
Public health programs	What are some of the public health programs? Please discuss the availability of potable water and sanitation in this community? What are some of the challenges associated with public health programs? Kindly discuss issues of waste management, immunization, and sensitization
NTD interventions	Please name some of the NTD interventions that are present in your community Have the interventions changed over time? Please discuss if the NTD interventions are adequate? Please, let me know what makes for a healthy community free from NTDs?

## Abbreviations

APOC: African programme for onchocerciasis control
CDI: Community directed intervention
CDT: Community directed treatment
CHVs: Community health volunteers
CLTS: Community-led total sanitation
FGs: Focus groups
FIISH: Financial inclusion improved sanitation and health
GWD: Guinea worm disease
ICM: Intensified case management
IPCHS: Integrated people-centred health services
KIs: Key informants
LF: Lymphatic filariasis
MDA: Mass drug administration
NGOs: Non-governmental organizations
NM: Not mentioned
NTDs: Neglected tropical diseases
PHC: Primary health care
PCT: Preventive chemotherapy
R&D: Research and development
SDGs: Sustainable development goals
SSA: Sub-Saharan Africa
STHs: Soil-transmitted helminths
UHC: Universal health care
WASH: Water, sanitation, and hygiene
WHA: World Health Assembly
WHO: World Health Organization
