# Supplementary material for: Local tips, global impact: community-driven measures as avenues of promoting inclusion in the control of neglected tropical diseases: a case study in Kenya
Source: Infect Dis Poverty. 2022 Aug 5;11:88. doi: 10.1186/s40249-022-01011-w (PMC9356398; doi:10.1186/s40249-022-01011-w)
Supplement: Supplementary file 1 — Additional file 1. Supplementary Tables and Appendix. [file 40249_2022_1011_MOESM1_ESM.pdf]

Legends to Supplementary Tables and Appendix

Supplementary Table 1:

The Table shows the current NTD programs in Kenya

Supplementary Table 2:

The Table shows the proposed NTD interventions from the communities

Supplementary Table 3:

The Table shows the proposed interventions from key informants

Supplementary Appendix A1:

Interview guide for collecting data on the capacity of local communities to address the burden of NTDs.

Results

The current NTD programs in Kenya

The most frequently mentioned and discussed factor by KIs and FGs was the school-based deworming program carried out by the Government of Kenya and its partners (Table 1).

Table 1. A Table showing the current NTD programs in Kenya

For example, the deworming program had decreased worm infestations and increased school participation.

> *'The Ministry of Health and Education coordinates the school health deworming activities which have really assisted children to increase their level of participation in schools because most cases of the worm infestations have reduced'* (Richard, male 38 years, KI).

The community members affirmed that deworming medication is distributed in schools. *'Our children get the medicines for worms in schools. It has been happening for some years now'* (Jacklyn, female 40 years, FGD).

Our data revealed that most counties adopted the Community-Led Total Sanitation program (CLTS) in Kenya. For example, the female community member specifically stated that: *In our community, the Ministry came with the CLTS program to prevent open defecation, and they said it also helps with reducing the transmission of worms.* (Jacklyn, female 40 years, FGD). While the KI below specifically explained the meaning, goal, and challenges of the CLTS program.

> *'My county is aiming to be 100% CLTS compliant, meaning everybody should have access to a toilet by the year 2020. I know there are relapses, here and there, because some have constructed and it has collapsed but that is another question all together'* (Richard, male 38 years, KI).

At the community level, the FG participants revealed that WASH interventions (Table 1), which included providing safe drinking water through chlorine filters installed in water collection kiosks, were commonly available as explicitly stated. 'We have *got WASH facilities[...] we have got water treatment at source and also point of use chlorine dispenser'* (Fatuma, female 30 years, FGD).

At the school level, we found that WASH interventions included personal hygiene, face washing, and the 'Unilever School of 5' as explicitly stated by the KI:

> *'We are using the Unilever School of 5. Unilever [corporate company] has for a long time been dealing with school health and WASH, focusing on handwashing at five critical times: when somebody wakes up, before eating, after eating, after using the toilet, after blowing*

your nose or coughing–5 critical times using soap. They have been providing soap and water facilities to encourage children to wash their hands 5 times a day, so we are riding on that approach, and we have established under the trachoma program, something we call Super School of 5' (Brian, male 50 years, KI).

Other ongoing programs (Table 1) included establishing and reviewing the National school health policy, which the Kis solely discussed. The National school health policy has eight thematic areas that guide WASH and deworming activities. Our analysis indicates that in 2018, Kenya embarked on a program assessment for onchocerciasis & human African trypanosomiasis (HAT) to begin the certification process for the two NTDs as stated explicitly by the KI:

'We are seeing that for conditions like onchocerciasis, which we officially said had been eliminated, we still don't have a concrete scientific basis to demonstrate elimination, so we are actually ensuring that we come up with processes that are going to demonstrate that yes, we do not have onchocerciasis and Human African trypanosomiasis' (Simon, male 40 years, KI).

The same KI further revealed that there is ongoing case finding activities for leprosy and hydatid disease, as well as plans to operationalize the NTD database to capture real-time disease prevalence:

'We have NTDs like hydatid disease[…], we are also looking at active case finding especially for leprosy, this one we just started so that we can pick up those cases. […] A big intervention is also data management. We have a lot of missing NTD data, and we are

trying to initialize data around NTDs and operationalize our NTD database' (Simon, male 40 years, KI*).*

Proposed interventions from the communities

Table 2: A Table showing the proposed NTD interventions from the communities

The community members discussed that counselling services and support groups for people infected with long-term disabling NTDs such as leprosy, elephantiasis, trachoma, and snake bites are lacking (Table 2). For example, this respondent noted that such support groups could be used to share information on what is expected of the conditions. '*It will also be nice to have government initiate support groups for people infected with NTDs, just like there are support groups for people having HIV/AIDs* (Anne, female 38years, FGD). Furthermore, '[…] the a*vailability of counselling programs for people infected with NTDs so that they do not die of stress from stigma* (John, male 47years, FGD).

Early physical examination was discussed especially for hydrocele patients. The participant specifically stated: *We as men also need to have physical examinations from time to time to capture the disease at an early stage'* (Fahim, male 30 years, FGD). Furthermore, the community members discussed proper diagnosis of NTDs in the health facilities (Table 2), and an adequate number of health care workers. *'We need proper diagnosis so that people get the treatment that is accurate. We also require adequate doctors in the health facilities so that we do not go very far for health services'* (Tina, Female 42 years FGD).

Community members proposed continuous sensitization to minimize ignorance and stated they would like to see more political goodwill from the county governments as an avenue for community empowerment.

'I would like as a member of this community to be sensitized because there are certain things, we can do on our own to prevent these diseases. We need to live in a clean environment, we need to be educated, not to stay out very late or even sleep outside like it is the norm with some of us because that is where the mosquitoes find and bite you' (Micah, male 52 years, FGD).

In terms of political goodwill, this participant had this to say:

'For me, I think there is a lot of laxity from the government. They keep asking us back and forth about our health concerns without any action, so for me, I see that there is a lack of seriousness even in terms of follow-up for NTDs (Tina, female 43 years, FGD).

Another recommendation was an improvement in WASH facilities, which included providing piped water, water treatment facilities, and the construction of latrines up to the household level. *'Another thing that brings the disease is unhygienic conditions. So, we have to have hygiene in our households. We need to have clean water and latrines'* (Linda, female 40 years, FGD).

At the health facility level, access to care and treatment (Table 2) was required free of charge or at an affordable rate as Kenya works towards providing UHC to its citizens; the present National Health Insurance Fund (NHIF), as it is, has remained unaffordable to many. *'For me, it would be that anyone who has an NTD can access treatment and care at an affordable price so that they can continue with their life and if possible, the treatment should be free'* (Fridah, female 45 years, FGD*).*

Proposed NTD interventions/policies from key informants

Table 3: A Table showing proposed interventions from key informants

Our findings revealed that NTD interventions coordinated by the Ministry of Health, the Ministry of Education, County governments, and other stakeholders need to be integrated (Table 3).

Our KIs proposed collaboration within the ministries and County governments and also between the ministries and the affected populations as noted by the respondent below:

> *'I feel there has to be an integration of all NTD stakeholders and other sectors including housing, agriculture, veterinary- they should all be brought on board so that they can know their role in this. An NTD-infected or affected person, they require food. Suppose we can bring in somebody who is an agriculturalist, who can tell them now that in Ganze[Constituency] you do not have rain. Can you plant this for your benefit? So, I feel that the interventions should be integrated. All other sectors are brought on board to address the problem of NTD'* (John, male 32 years, KI).

There is a need to engage communities through capacity building around NTD control. This should involve forming active community committees comprising health workers, CHVs, and individuals in the community for health education and NTD surveillance:

> *'I think one important area we need to take is a bottom-up approach and not thinking for the community but also getting the community to give their ideas especially when we are formulating this policy, engaging the communities to give their perception on what policy and what they want these policies to address'* (Mark, male 50 years, KI).

The KIs suggested that legislative acts (Table 3) around the treatment of NTDs need to be enforced for highly infectious NTDs. For example, Section 84 of the Public Health Act CAP 242

states that persons receiving treatment for leprosy need to be followed up to ensure that they adhere to the treatment regimen as stated below:

> *'If we can borrow and understand the Constitution of Kenya, that everybody has a right to life, everyone has a right to health, then it can be emphasized, and everyone sensitized to adhere to their treatment regimen. We have Public Health CAP 242, which focuses on those people suffering from infectious diseases such as leprosy. There is an intervention that if a person does not come to take medication, by law, we have a right to follow and assist that person in taking up medication'* (Simon, male 40 years, KI).

Similarly, it was suggested that NTDs need to be included in the school and public health curriculum for sensitization and behavioural change among young adults:

> *'I think the education of diseases and neglected diseases, for instance, needs to be part of the curriculum for students. I think we should have an integrated curriculum that is able to take all this NTD information and put it together in a package that can actually be delivered to the students and to the communities holistically and cheaply and much more efficiently'* (Tom, male 41 years, KI).

Individuals suffering from long-term debilitating and disabling NTDs such as leprosy, elephantiasis, trachoma, and snake bites require counselling services and support groups (Table 3) to help them deal with the psychosocial effects of NTDs as specifically mentioned below:

> *'Because like HIV/AIDS they have a social group and they know each other, and they support each other just like diabetes they know each other. But for elephantiasis or leprosy, we have not started such as a county/country. Maybe we can, we can think of that as we have launched the MMDP, Morbidity Management and Disability Prevention Program.*

Maybe we can think of that so the people within a certain area can be meeting to socially support each other and give advice on the things they are supposed to be doing for example, how they are supposed to take care of their swollen legs' (Mark, male 50 years, KI).

The KIs discussed an improvement in vaccination services, with NTD vaccines such as rabies being part of the Kenya Expanded Programme on Immunization (KEPI) rather than on-demand vaccines when dog bites occur:

'If I were directly involved in the passing of health policies for NTDs, I would move the rabies vaccines from being an on-demand vaccine and make it a part of the expanded program of immunization[compulsory vaccines]. The reason for this is that a child needs not to have money to get the measles vaccine; I think a family needs not to have money to be saved from the deadly rabies virus. So it is moving those vaccines to be completely free because they are lifesaving and they are too expensive for the people that are marginalized. Number two thing is that I would put money to eliminate the disease at the source. In fact, rather than spend millions of shillings buying the vaccine on the human side, I would spend a bit of that money buying vaccines on the animal side and making sure that they are well funded because if they remove the disease in the dogs, then the humans are safe and it is cheaper, actually much cheaper' (Tom, male, 41 years, KI).
